# Supplementary figures and images for: Data-driven learning of structure augments quantitative prediction of biological responses
Source: PLoS Comput Biol. 2024 Jun 3;20(6):e1012185. doi: 10.1371/journal.pcbi.1012185 (PMC11233023; doi:10.1371/journal.pcbi.1012185)

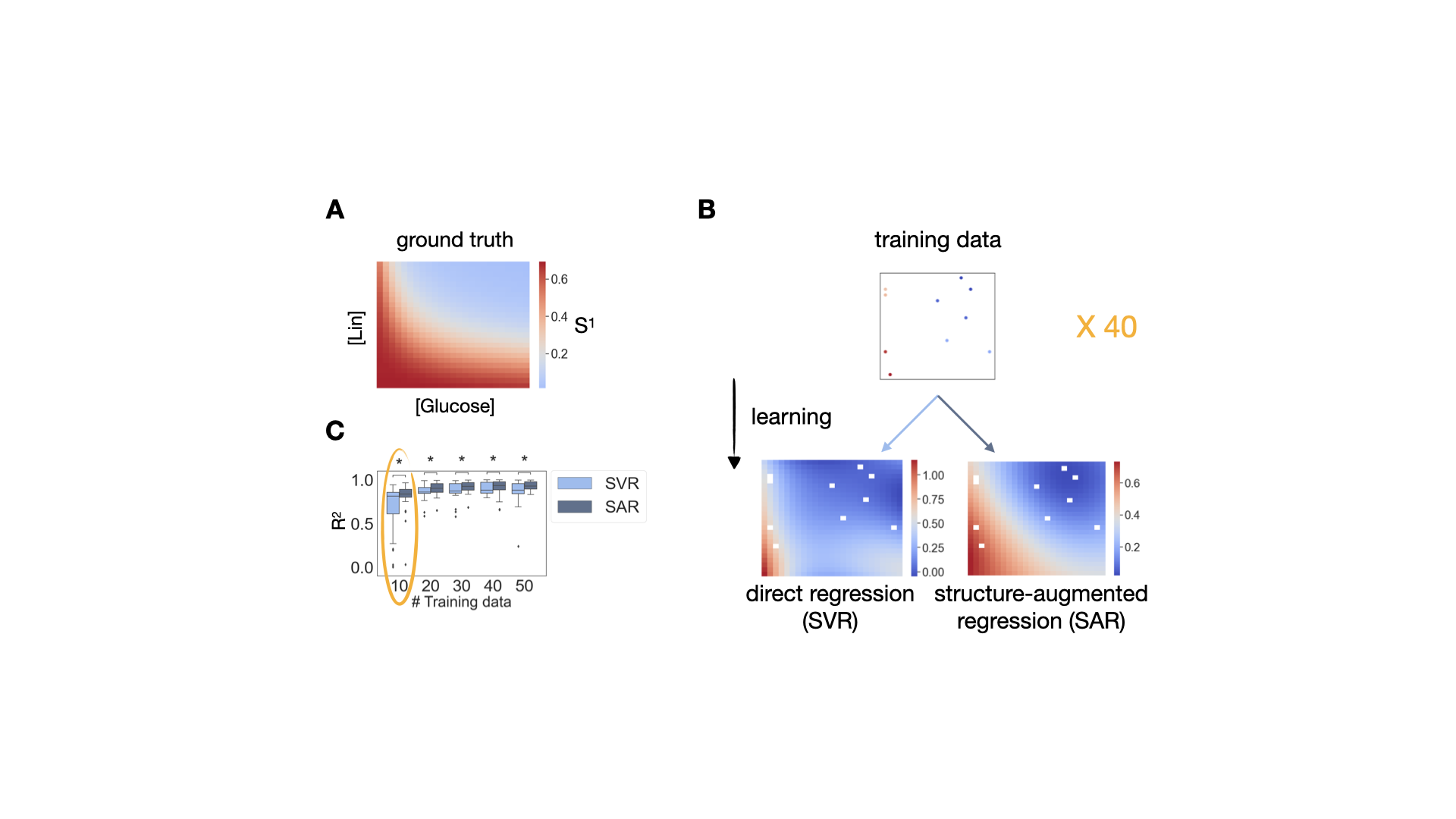

Supplement: S1 Fig — A. The ground truth to demonstrate the flow of training and testing procedure. The x- and y- axis represent two environmental factors that can be tuned to control the growth of some biological system: in this case, glucose and linoleic acid (Lin). The heatmap color represents the final cell density of that system. B. The training and testing procedure. We first split the ground truth into training and testing sets. Note the small size of training set corresponds to the limited amount of experimental data usually available. We then train the traditional regression model (direct regression pipeline) and our structure-augmented regression (SAR) model on the same training set simultaneously. The two learned models will then be applied to the same testing set for prediction. The traditional regression pipeline is represented by the light blue arrows and the structure-augmented regression pipeline is represented by the dark blue arrows throughout this figure and all other figures in the manuscript. The prediction accuracy is evaluated using R2 measurements. Here we just show one set of 10 training data. The “X40” means we carry out the same training and testing procedure 40 times on 40 different training and testing set splits. We then compare the 40 pairs of R2 using the Mann-Whitney statistical test. This gives us the pipeline performance comparison on one specific training data amount, highlighted in orange in C. C. For each of the 10, 20, 30, 40 or 50 specific amount of training data, we carry out the same type of pipeline comparison shown in B. This gives us a more comprehensive view of method performance across a wide range of training data availability. p-value annotation legend: ns: 0.05 < p < = 1.0; *: 0.01 < p < = 0.05; **: 0.001 < p < = 0.01; ***: 0.0001 < p < = 0.001; ****: p< = 0.0001. (TIFF) [file pcbi.1012185.s001.tiff]

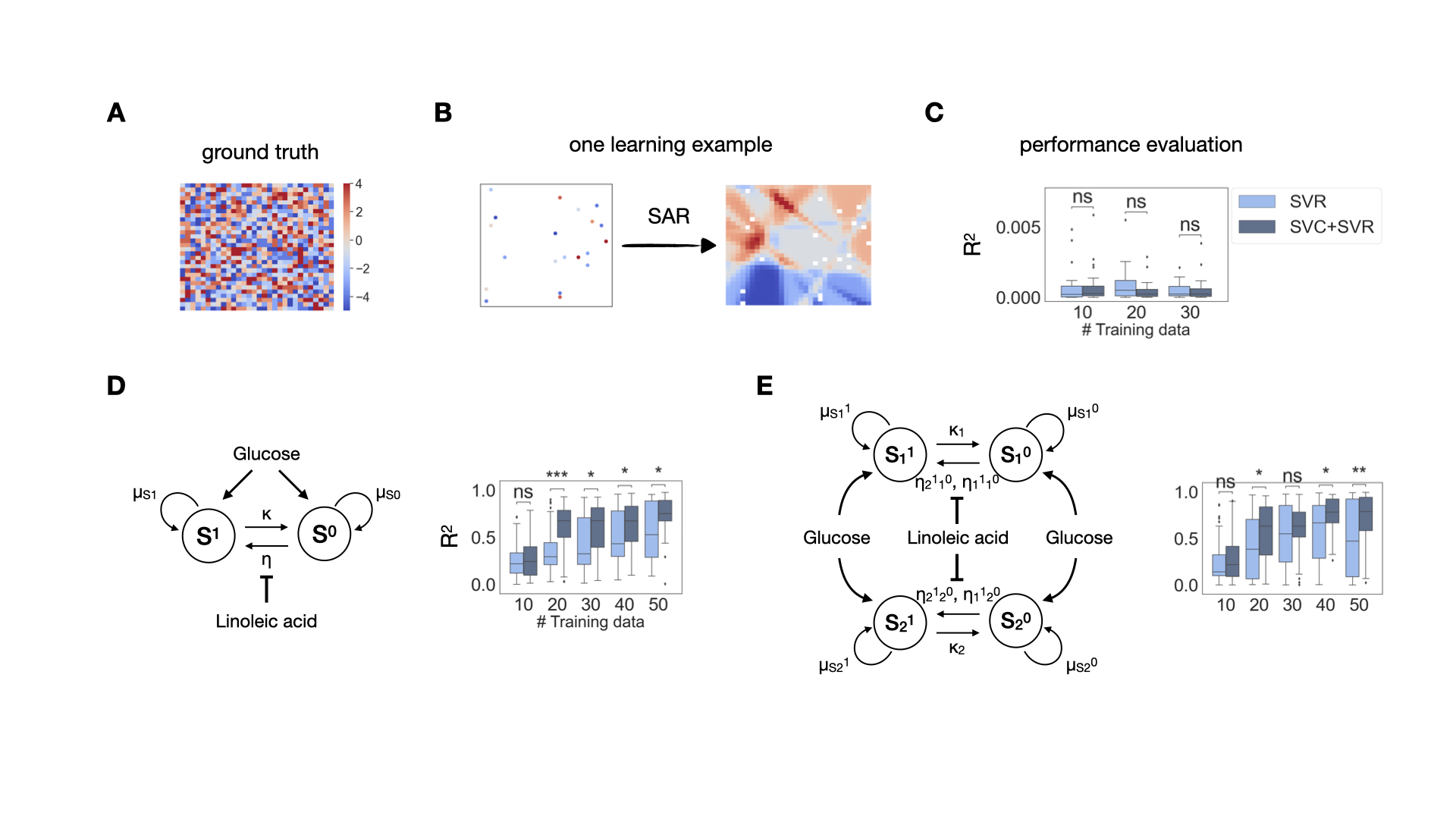

Supplement: S2 Fig — A. Ground truth of a synthetic random landscape. B. One learning example of the random landscape. The learning algorithm struggles to learn any structure. C. The method comparison of the two methods. The performance evaluation is done in the same way as in S1 Fig. The two methods perform equally poorly on a landscape with no structure. D. Method comparison on the community of one species and one plasmid as in Fig 2A. Structure-augmented regression consistently outperforms starting from with a training data set of 20 points and shows less variance. E. Method comparison on the community of two species and one plasmid as in Fig 2D. Structure-augmented regression consistently outperforms starting from with a training data set of 20 points and shows less variance as well. p-value annotation legend: ns: 0.05 < p < = 1.0; *: 0.01 < p < = 0.05; **: 0.001 < p < = 0.01; ***: 0.0001 < p < = 0.001; ****: p< = 0.0001. (TIFF) [file pcbi.1012185.s002.tiff]

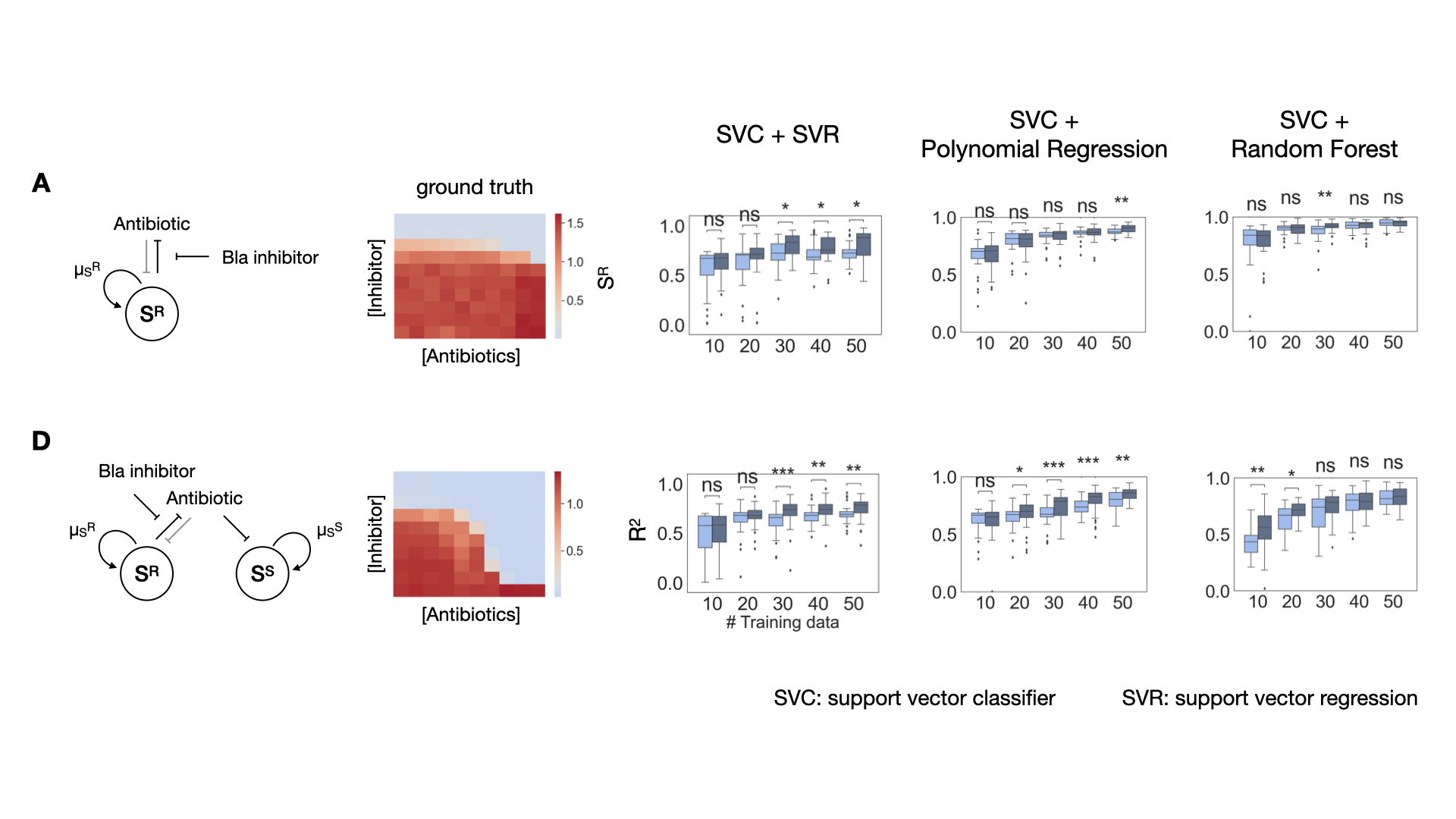

Supplement: S3 Fig — Statistical tests of the regression and structure-augmented regression methods on the two communities in Fig 3. The first row corresponds to the example in Fig 3A–3C. The second row corresponds to the example in Fig 3D–3F. Here we include three different kinds of regression methods: SVR, polynomial regression and random forest regression. The layout of the two rows (A and B) are the same. The first panel from left: schematic of the community. The second panel: ground truth of final population density. The next three panels: method comparisons using flexible ML pipelines. We can see that structure-augmented regression consistently outperforms the regression itself for all these three regression methods. The improvement for the first dataset is not as obvious, since that the landscape itself in this sample is very simple. p-value annotation legend: ns: 0.05 < p < = 1.0; *: 0.01 < p < = 0.05; **: 0.001 < p < = 0.01; ***: 0.0001 < p < = 0.001; ****: p< = 0.0001. (TIFF) [file pcbi.1012185.s003.tiff]

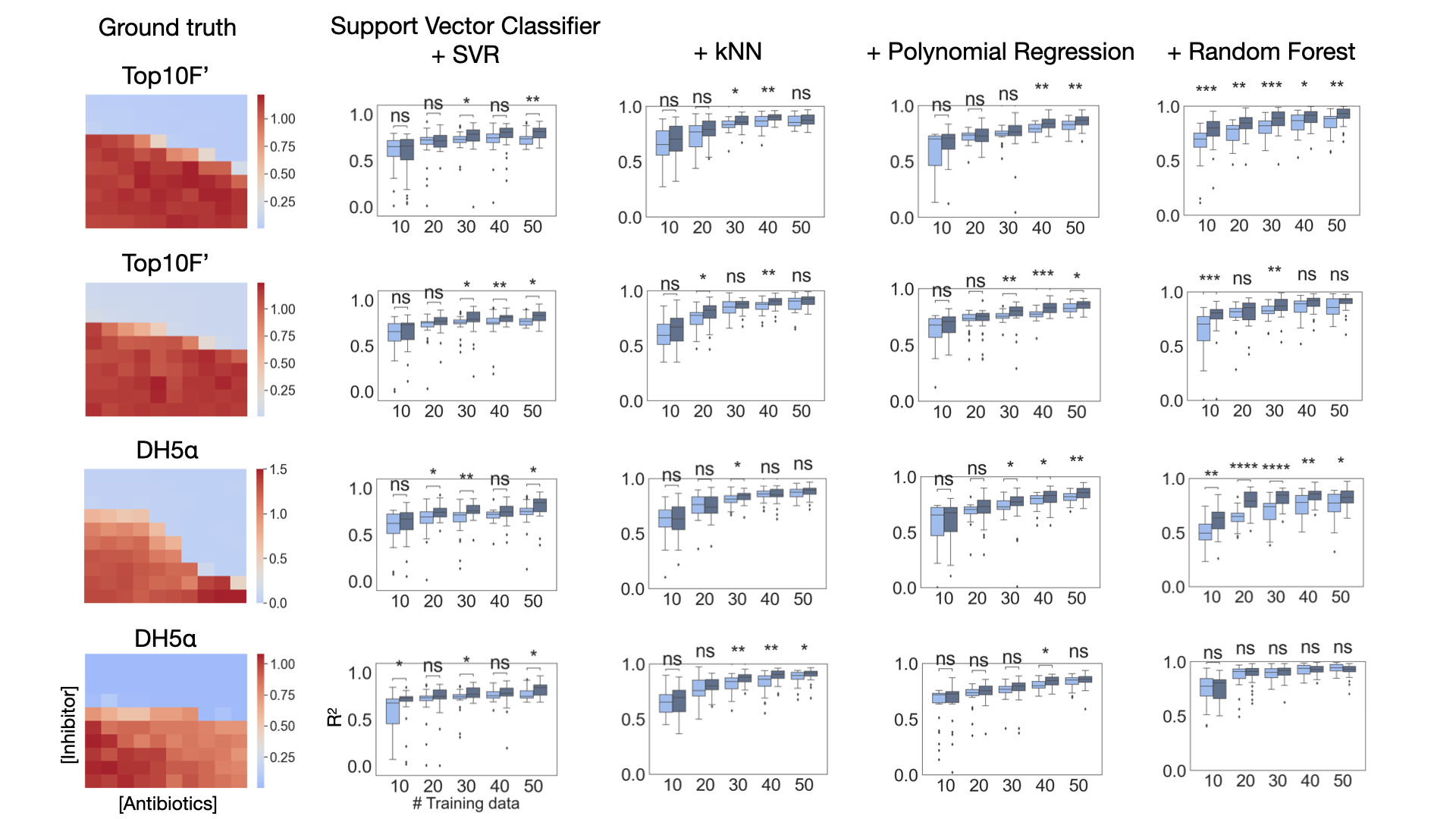

Supplement: S4 Fig — All experimental data here are still the final cell density of β-lactam resistance communities, under combination treatments. The communities are either DH5α or Top10F’ E.coli cells, shown as labels on top of each ground truth panel. All combinations use two drugs: one antibiotic and one β-lactamase inhibitor. Each row represents application on one specific experimental result. The layout of each row is the following: Left panel: ground truth of final population density. The next four panels: method comparisons using flexible ML pipeline that integrates various regression methods with SVC, including SVR, KNN, polynomial regression and random forest regression. We can see that when applying to landscapes of different types of structures, our method consistently improves the prediction accuracy. p-value annotation legend: ns: 0.05 < p < = 1.0; *: 0.01 < p < = 0.05; **: 0.001 < p < = 0.01; ***: 0.0001 < p < = 0.001; ****: p< = 0.0001. (TIFF) [file pcbi.1012185.s004.tiff]

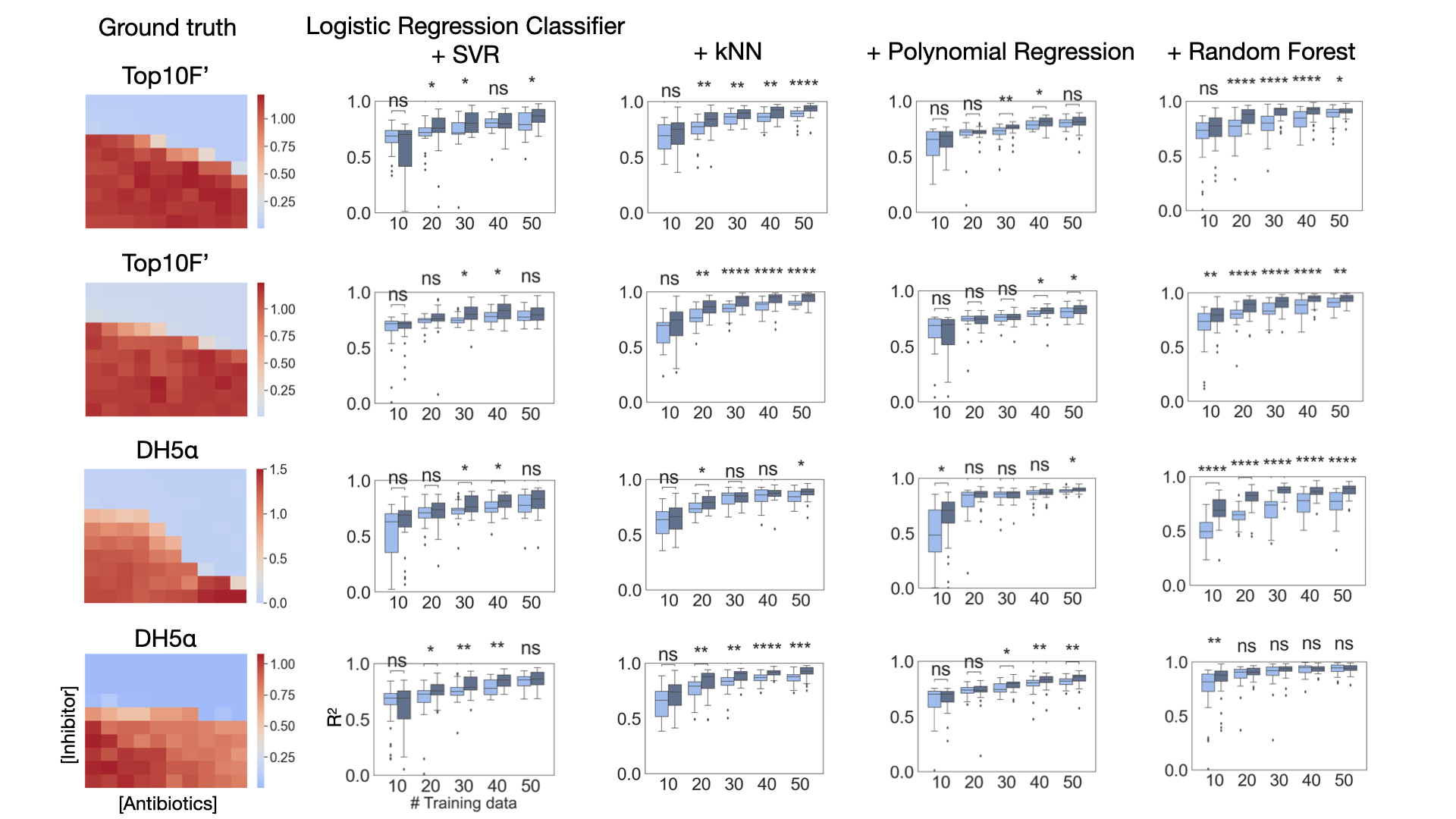

Supplement: S5 Fig — All experimental data here are the same as in S4 Fig (still the final cell density of β-lactam resistance communities, under combination treatments). The communities are either DH5α or Top10F’ E.coli cells, shown as labels on top of each ground truth panel. All combinations use two drugs: one antibiotic and one β-lactamase inhibitor. Each row represents application on one specific experimental result. The layout of each row is the following: Left panel: ground truth of final population density. The next four panels: method comparisons using flexible ML pipeline that integrates various regression methods with logistic regression classifier, including SVR, KNN, polynomial regression and random forest regression. We can see that when applying to landscapes of different types of structures, our method with logistic regression classifier in the first step also consistently improves the prediction accuracy. p-value annotation legend: ns: 0.05 < p < = 1.0; *: 0.01 < p < = 0.05; **: 0.001 < p < = 0.01; ***: 0.0001 < p < = 0.001; ****: p< = 0.0001. (TIFF) [file pcbi.1012185.s005.tiff]

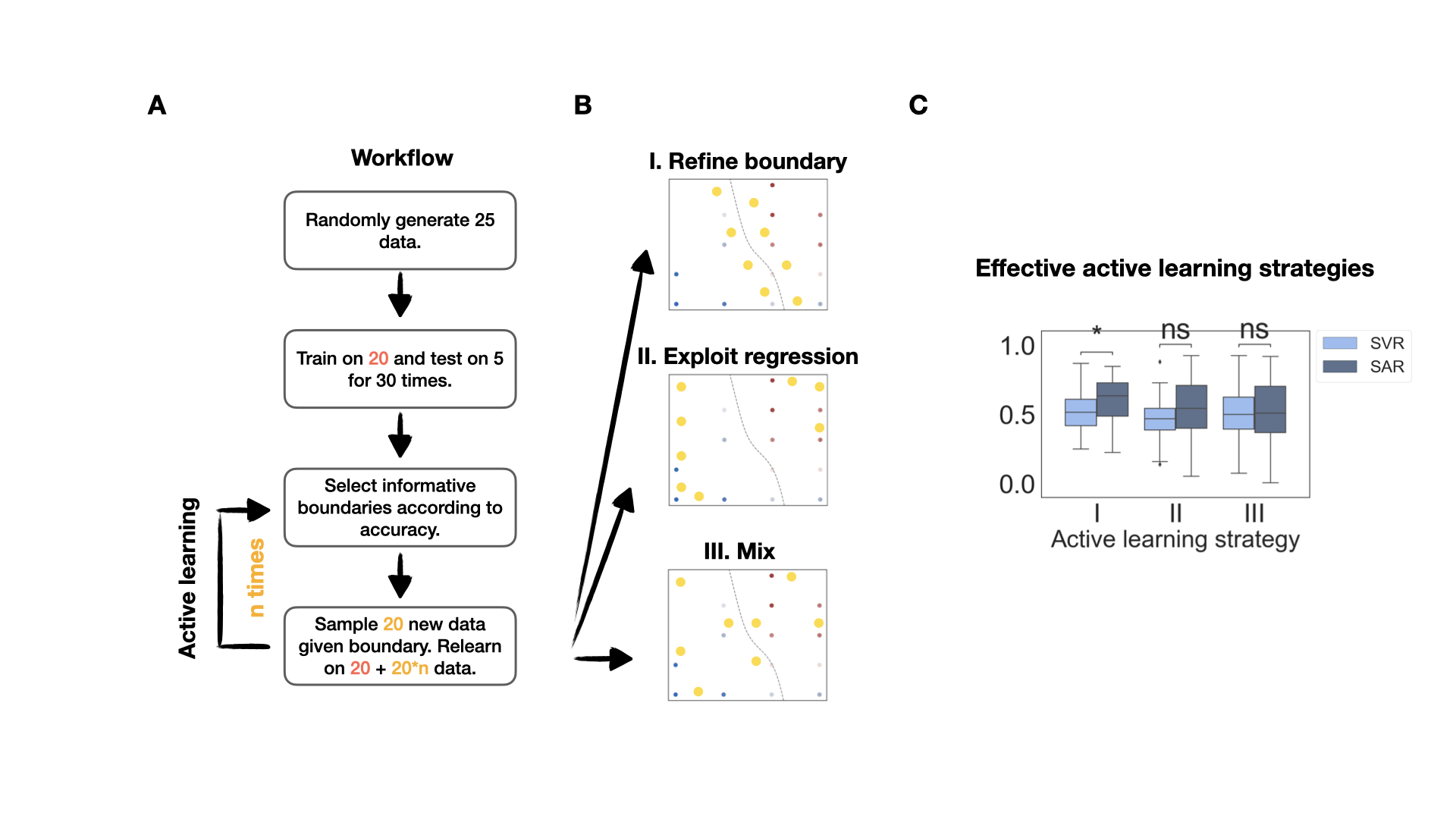

Supplement: S6 Fig — A. The four-step process of combining structure-augmented regression and active learning. The key is to utilize the learned structure in future rounds of experimental data generation after the first round of learning. After experimentally generating 20 data, the first step is to apply the pipeline on them as usual to learn 30 different structures by learning on 30 different training sets. The second step is to pick the top informative boundaries out of the 30 learned ones by final prediction accuracy improvement of SAR over SVR. The boundary that contributes to the most improvement is the most informative. The third step is to generate the next round of experimental data based on the best boundary. Fourth, the ML pipeline is applied on the data generated in both rounds. If needed, the number of iterations, n, can increase for further actively learn. To achieve statistically significant comparison, in our test, we pick the boundaries that contribute to an increase of R2 value > 0.1, sample new data around each of these boundaries and relearn, then compare two sets of new R2 values. B. Cartoon for three types of combination schemes. Assume that the dashed black line in the middle of the scatter plot is the learned boundary from the first round of learning, there are three combination schemes given this information. There are three schemes of the new data selection: refining boundary by selecting data around the boundary; exploiting regression by selecting data away from the boundary; combining the advantages of classification and regression by combing these two approaches. C. Second round of experiments needs to further refine learned structure. There are three schemes of the new data generation based on the best learned structure of the first round: refining boundary by selecting more data around the boundary; exploiting regression by selecting data away from the boundary; combining the advantages of these two strategies. Running the same algorithm on new data genera [file pcbi.1012185.s006.tiff]

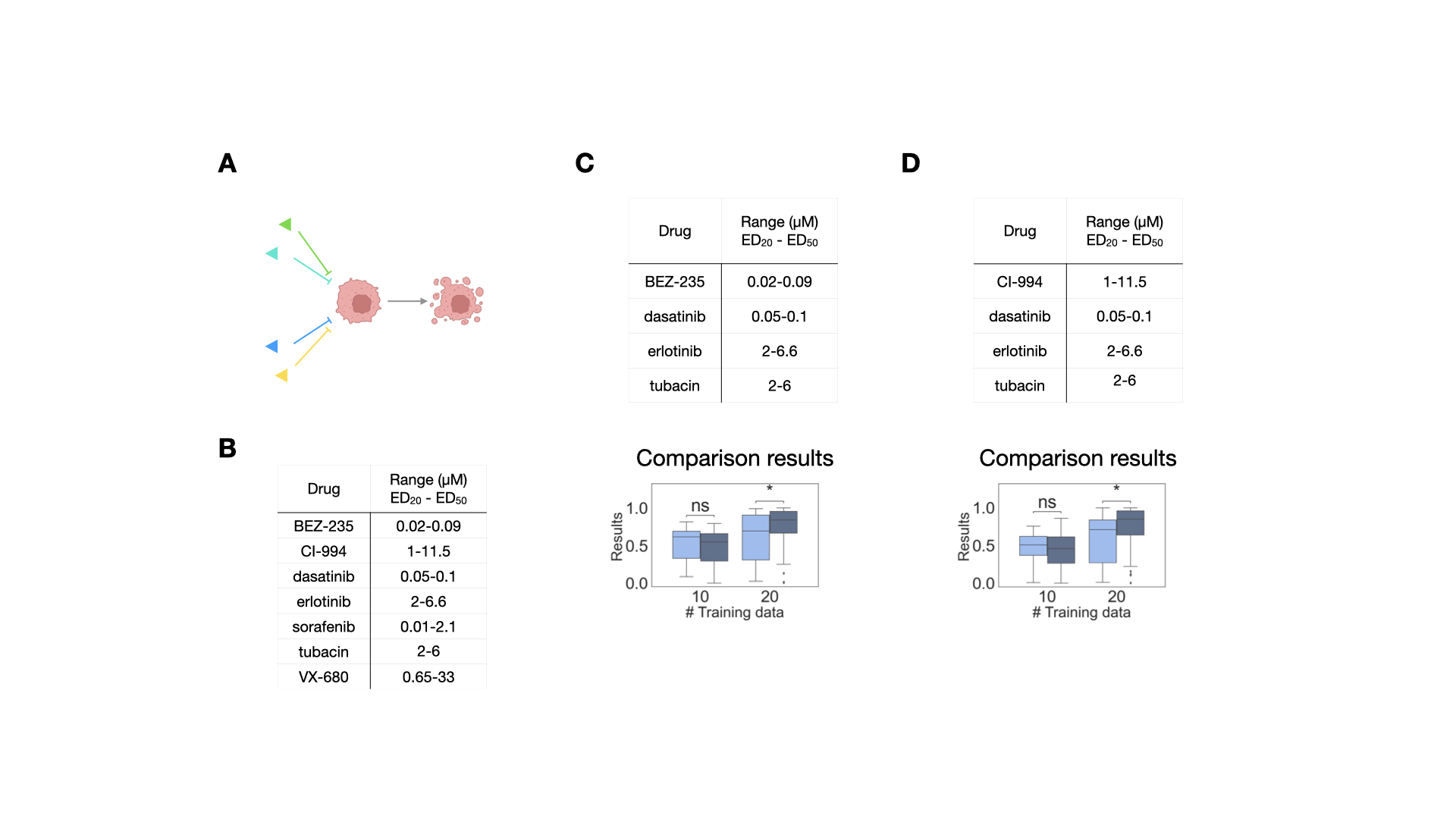

Supplement: S7 Fig — A. Illustration of applying four different cancer drugs for better treatment results (created with BioRender.com). B. The seven drugs and their tested range for Fig 5A. (C, D.) Applications on two different 4-drug combination treatments of cancer cell-line 786-O. Both figures follow the same format. The top table contains the drug and dosage information; the bottom figure is the method comparison results. Both experiments generated 25 datapoints in total, so the method comparison only trained on either 10 or 20 data and tested on the rest. The structure-augmented regression consistently outperforms direct regression while being trained on 20 datapoints. (TIFF) [file pcbi.1012185.s007.tiff]
