# Supplementary material for: Data-driven learning of structure augments quantitative prediction of biological responses
Source: PLoS Comput Biol. 2024 Jun 3;20(6):e1012185. doi: 10.1371/journal.pcbi.1012185 (PMC11233023; doi:10.1371/journal.pcbi.1012185)
Supplement: Attachment — Submitted filename: 2023-Review_PCOMPBIOL-D-23-01992.pdf [file pcbi.1012185.s008.pdf]

Summary & Overall Impression

In this manuscript the authors propose a new machine learning method, *Structure-Augmented Regression* (SAR), that combines classification and regression to predict the response of biological systems to perturbations of multiple system parameters when only a few data points are available and evaluate it on both synthetic and experimental data. In addition, the authors design an active learning framework based on SAR.

The manuscript addresses the important methodological challenge of applying machine learning methods when data is scarce, e.g. due to the combinatorial explosion of possible experimental conditions. The authors approach this problem in an interesting and original way. They conduct sufficient computational experiments to showcase the strengths and weaknesses of their approach. While the results are presented in a clear and accessible way, the method itself could be explained in more detail, especially the parts that pertain to active learning. In my opinion, this would greatly improve the manuscript's usefulness for the community.

Overall, I think the manuscript makes a valuable contribution to the literature that will be of interest to a broad audience. However, addressing the following major and minor issues could, in my opinion, further improve the manuscript.

Major Issues

- Various passages suggest that SAR is a method that incorporates *constraints*, derived from the decision boundary of a classifier, into a regression algorithm, e.g. "impose the learned boundary onto the subsequent regression analysis, as a constraint" (p. 7), "our soft-constrained regression method" (p. 7) and "result of the soft structural constraint" (p. 9). While thinking about the decision boundary as a constraint can be a useful heuristic, I wonder if this is technically correct, as the distance from the decision boundary is used as an additional input feature in the regression and does not *constrain* the output of the regression. An argument could be made in the special case of support vector regression, as features do enter as constraints into the primal optimization problem, but I don't see why this would be the case for other types of regression. I would appreciate it if the authors could describe their reasons for viewing SAR as a constraint regression instead of for example a feature engineering technique.

- As the main contribution of the manuscript is in my view a methodological one, a more detailed methods section would strengthen the work and make it easier for readers to adopt the proposed algorithm in their own analyses:

    - Equation (10) includes a kernel but it is not clear which kernel the authors used and whether the kernel itself or kernel hyperparameters were optimized (e.g. with cross-validation). Related to this, it would be interesting to comment on the interpretation of the quantity *f(p)* when a non-linear kernel is used.
    - I found the application of SAR to active learning very interesting and was surprised to find no description of it in the methods section and only a brief one in the supplementary information. This part deserves proper treatment in the methods section. In particular, the three strategies (*refine boundary, exploit regression,* and *mix*) need to be defined more rigorously and in sufficient detail to allow readers to implement them.

- The *Discussion* section already includes some of the method's limitations (e.g. "determining critical decision boundaries will involve some trial and error" p. 20) but I think it would be

important to explain further in which cases SAR can and cannot be applied and to point out the assumptions underlying the classification and regression algorithms more clearly. To give an example, the current version of the algorithm appears to be restricted to the case of binary classification as it uses a simple support vector machine. Discussing these limitations could also be a natural starting point to comment on possible next steps to improve the method.

Minor Issues & Comments

- In all heatmaps throughout the manuscript, I do not fully understand the choice and centering of the color scale. Do the red and blue regions reflect the different classes? If so, shouldn't the gray part of the color scale correspond to the threshold concentration (e.g. 0.2 in Fig. 1), chosen by the authors to delineate the classes?
- The authors sometimes write *structure-augmented* and sometimes *structure-assisted* regression. For the sake of consistency, it would be good to choose one or the other.
- This may to a certain extent be a question of style and personal preference but in most figures axis labels are only present for a subset of the plots and (supposedly) apply to other plots within the same figure. I understand the author's intent not to clutter the figures with too many labels but I found it at times cumbersome to find the right label for a given axis. Labeling each axis individually may indeed be too much, but I think repeating the labels unless plots are directly stacked will make the figures easier to parse.
- In the section *Learnt structure contains rich information to assist regression* (p.6, first paragraph), I would suggest including a reference to the differential equations in the methods section (and the simulation parameters). This would make it easier for a mathematically mature but biologically less well-versed audience to follow the text.
- In the section *Application of structure-assisted regression on simulation data* when presenting the computational experiment with four different populations, the authors speak of "two boundaries" (p.9), which makes sense visually. I see, however, some potential for confusion here as one might ask what the distance from which boundary SAR actually uses or how information from both boundaries is combined in SAR. I acknowledge that this becomes clear later on with the mathematical description provided in the *Methods* section but I encourage the authors to address this early on and more explicitly.
- In the section *SAR improves prediction on experimental data*, I was pleased to see *SAR* tested with regression algorithms beyond support vector regression. Unfortunately, the comparison is mentioned only very briefly in the main text. One should of course be careful not to overinterpret the results, but formulating some hypothesis about the origin of the differences between regression algorithms in *Supplementary Figure 3* could be insightful, e.g. why do some regression algorithms seem to incorporate the additional information from the classification step more easily than others?
- The brief description of active learning ("the general scheme of such ML-assisted guidance is called 'active learning'." p. 13), conveys the general idea well but it would be good to provide some references to reviews on the topic of active learning. I believe that this would not only be useful for readers who are less familiar with the topic but also help to locate the author's contribution in the research landscape.

- In the *Discussion* section, I struggle to understand the following passage “[...] provide a fundamentally different approach by exploiting the data itself instead of the method” (p. 19). While I welcome the author’s efforts to distinguish their method clearly from ensemble approaches, I’m not sure in which sense an ensemble exploits “the method” (p. 19), whereas SAR would “exploit[...] the data” (ibid.).

- On pages 24 and 25, I would suggest changing “transforms the data into a linearly separable space” in a way that reflects the fact that linear separability is a property of two sets of points not of a space.

- On pages 25 and 26, the authors state “a soft constraint is applied to the additional feature from classification, *f(p’)*, by letting the regression method learn an optimal weight *w* for it [...]”. As far as I understand the weight *w*, is a hyperparameter that is set using cross-validation. I advocate reserving the word *learn* for parameters that are set by the learning algorithm and not during hyperparameter tuning.
