# Supplementary material for: Data-driven learning of structure augments quantitative prediction of biological responses
Source: PLoS Comput Biol. 2024 Jun 3;20(6):e1012185. doi: 10.1371/journal.pcbi.1012185 (PMC11233023; doi:10.1371/journal.pcbi.1012185)
Supplement: Attachment — Submitted filename: Reviewer Comments Response.docx [file pcbi.1012185.s009.docx]

**Point-by-point responses.**

**Reviewer #1:**

**Summary & Overall Impression:**

In this manuscript the authors propose a new machine learning method, Structure-Augmented Regression (SAR), that combines classification and regression to predict the response of biological systems to perturbations of multiple system parameters when only a few data points are available and evaluate it on both synthetic and experimental data. In addition, the authors design an active learning framework based on SAR.

The manuscript addresses the important methodological challenge of applying machine learning methods when data is scarce, e.g. due to the combinatorial explosion of possible experimental conditions. The authors approach this problem in an interesting and original way. They conduct sufficient computational experiments to showcase the strengths and weaknesses of their approach. While the results are presented in a clear and accessible way, the method itself could be explained in more detail, especially the parts that pertain to active learning. In my opinion, this would greatly improve the manuscript’s usefulness for the community.

Overall, I think the manuscript makes a valuable contribution to the literature that will be of interest to a broad audience. However, addressing the following major and minor issues could, in my opinion, further improve the manuscript.

We thank the reviewer for the thorough review of the manuscript, for recognizing the originality of the work, as well as its potential value to the research community. We also appreciate all the constructive comments, which we have thoroughly addressed below and in our revised manuscript.

**Major Issues:**

1. Various passages suggest that SAR is a method that incorporates constraints, derived from the decision boundary of a classifier, into a regression algorithm, e.g. “impose the learned boundary onto the subsequent regression analysis, as a constraint” (p. 7), “our soft-constrained regression method” (p. 7) and “result of the soft structural constraint” (p. 9). While thinking about the decision boundary as a constraint can be a useful heuristic, I wonder if this is technically correct, as the distance from the decision boundary is used as an additional input feature in the regression and does not constrain the output of the regression. An argument could be made in the special case of support vector regression, as features do enter as constraints into the primal optimization problem, but I don’t see why this would be the case for other types of regression. I would appreciate it if the authors could describe their reasons for viewing SAR as a constraint regression instead of for example a feature engineering technique.

We regret the confusion. As noted by the reviewer, the structural information is indeed a feature extracted from the data, during the classification step.

During regression, this feature is encoded as the distance between each point and the boundary, learned from the classification step. That is, during regression, each input data point is represented by two sets of features: (1) the combination of input concentrations and, (2) the distance of this combination to the boundary. As such, regardless of the choice of the downstream model, the structural information will indeed affect the regression outcome.

In contrast, in standard regression, only the input concentrations are used as inputs.

We have revised the manuscript accordingly to clarify this point.

2. As the main contribution of the manuscript is in my view a methodological one, a more detailed methods section would strengthen the work and make it easier for readers to adopt the proposed algorithm in their own analyses: Equation (10) includes a kernel but it is not clear which kernel the authors used and whether the kernel itself or kernel hyperparameters were optimized (e.g. with cross-validation). Related to this, it would be interesting to comment on the interpretation of the quantity f(p) when a non-linear kernel is used.

We thank the reviewer for making this suggestion and we have revised the Methods section accordingly.

Specifically, during the training process, we let SVC choose the best kernel from linear, polynomial and RBF kernels. Likewise, the kernel hyperparameters, i.e., the degree and the coefficient term for the polynomial kernel, the gamma term for both polynomial and RBF kernels, are all optimized with cross-validation. We used python’s scikit packages for all training and optimization.

Regardless of the kernels used, the resulting f(p) values have a monotonical dependence on the distance to the boundary in the transformed space by the kernel trick. Empirically, we found this monotonic relationship between f(p) and distance in the transformed space could assist the regression prediction by providing useful information.

We have added this part in the method section as well.

3. I found the application of SAR to active learning very interesting and was surprised to find no description of it in the methods section and only a brief one in the supplementary information. This part deserves proper treatment in the methods section. In particular, the three strategies (refine boundary, exploit regression, and mix) need to be defined more rigorously and in sufficient detail to allow readers to implement them.

We thank the reviewer for recognizing that the application of SAR to active learning is interesting, and we have revised the Methods section accordingly to add in the “application of SAR to active learning” section.

4. The Discussion section already includes some of the method’s limitations (e.g. “determining critical decision boundaries will involve some trial and error” p. 20) but I think it would be important to explain further in which cases SAR can and cannot be applied and to point out the assumptions underlying the classification and regression algorithms more clearly. To give an example, the current version of the algorithm appears to be restricted to the case of binary classification as it uses a simple support vector machine. Discussing these limitations could also be a natural starting point to comment on possible next steps to improve the method.

We appreciate the suggestion. We have made the discussion on the limitations and potential future extension of this method more comprehensive.

We mentioned a key limitation of this method in the first result section and supplementary figure two. When the response surface has no distinctive structure or the structure is too complicated, it would be difficult to identify clear boundaries from limited data. As such, imposing a structural constraint could be counter-productive. However, for biological examples we analyzed, we found that their response landscapes all exhibit distinctive, sufficiently simple structure features, where our method is effective.

We also added potential future steps on extension this method to multi-class classifications. Specifically, one can use either one-against-all or one-against-one SVC methods to discover multiple boundaries that could be used to capture the underlying quantitative data trend^1,2^.

**Minor Issues & Comments:**

1. In all heatmaps throughout the manuscript, I do not fully understand the choice and centering of the color scale. Do the red and blue regions reflect the different classes? If so, shouldn’t the gray part of the color scale correspond to the threshold concentration (e.g. 0.2 in Fig. 1), chosen by the authors to delineate the classes?

We thank the reviewers for pointing this out.

Indeed, for the SAR pipeline the two colors should reflect the different classes and we have changed the center point on these heatmaps accordingly.

2. The authors sometimes write structure-augmented and sometimes structure-assisted regression. For the sake of consistency, it would be good to choose one or the other.

We thank the reviewer for pointing this out and agree that the interchange of these two phrases can be confusing. We have decided to use “structure-augmented regression” throughout and have changed the manuscript accordingly.

3. This may to a certain extent be a question of style and personal preference but in most figures axis labels are only present for a subset of the plots and (supposedly) apply to other plots within the same figure. I understand the author’s intent not to clutter the figures with too many labels but I found it at times cumbersome to find the right label for a given axis. Labeling each axis individually may indeed be too much, but I think repeating the labels unless plots are directly stacked will make the figures easier to parse.

We acknowledge that the current sparse labeling can be confusing, especially in Fig1-3, and we have added a few more labels to make them clearer.

4. In the section Learnt structure contains rich information to assist regression (p.6, first paragraph), I would suggest including a reference to the differential equations in the methods section (and the simulation parameters). This would make it easier for a mathematically mature but biologically less well-versed audience to follow the text.

We thank the reviewer for this thoughtful comment and have added a couple of citations where these models are used to study systems biology^3,4,5^.

5. In the section Application of structure-assisted regression on simulation data when presenting the computational experiment with four different populations, the authors speak of “two boundaries” (p.9), which makes sense visually. I see, however, some potential for confusion here as one might ask what the distance from which boundary SAR actually uses or how information from both boundaries is combined in SAR. I acknowledge that this becomes clear later on with the mathematical description provided in the Methods section but I encourage the authors to address this early on and more explicitly.

We thank the reviewer for their thorough evaluation.

We have explained this part more carefully in the main result section.

6. In the section SAR improves prediction on experimental data, I was pleased to see SAR tested with regression algorithms beyond support vector regression. Unfortunately, the comparison is mentioned only very briefly in the main text. One should of course be careful not to overinterpret the results, but formulating some hypothesis about the origin of the differences between regression algorithms in Supplementary Figure 3 could be insightful, e.g. why do some regression algorithms seem to incorporate the additional information from the classification step more easily than others?

We thank the reviewer for the astute observation. We do not have an intuitive explanation on the observed difference based on the inner workings of different algorithms. However, it appears that the difference might result from the baseline performance of an algorithm on limited data. If the regression by a particular algorithm performs poorly, there is more room for further improvement, either by generating more data or by using structure-augmented regression. However, if an algorithm is already performing well, incorporation of the structural information may not help as much.

For example, in the first example (first row), SVR alone performs the worst across all three regression methods, and structure-augmented SVR shows the most improvement. However, structure-augmented polynomial regression and structure-augmented random forest does not show as much improvement since there isn’t much to improve for these two methods to start with. On the other hand, in the second example (second row), both SVR and polynomial regression alone does a poor job, so structure-augmented SVR and structure-augmented polynomial regression show much more significant improvements than structure-augmented random forest.

We observe similar trends when using other algorithms (Supplementary Figure 4).

Specifically, in the third example (third row), random forest regression alone performs the worst across all four regression methods, and structure-augmented random forest regression shows the most improvement. However, structure-augmented kNN and structure-augmented polynomial regression are on par with the structure-augmented random forest regression, yet the improvement is not as significant since there is not as much to improve for these two methods. On the other hand, in the fourth example (last row), random forest regression alone performs well already. Therefore, while structure-augmented random forest regression still improves the performance, there is not much room to improve. Meanwhile, all other three regression methods alone perform worse, so structure-augmented SVR, kNN, and polynomial regression all show more significant improvement.

7. The brief description of active learning (“the general scheme of such ML-assisted guidance is called ‘active learning’.” p. 13), conveys the general idea well but it would be good to provide some references to reviews on the topic of active learning. I believe that this would not only be useful for readers who are less familiar with the topic but also help to locate the author’s contribution in the research landscape.

We thank the reviewer for this suggestion and have added a few active learning review references^6,7,8^ at the beginning of the active learning section.

8. In the Discussion section, I struggle to understand the following passage “[...] provide a fundamentally different approach by exploiting the data itself instead of the method” (p. 19). While I welcome the author’s efforts to distinguish their method clearly from ensemble approaches, I’m not sure in which sense an ensemble exploits “the method” (p. 19), whereas SAR would “exploit [...] the data” (ibid.).

We regret the confusion.

The ensemble methods apply different ML regression methods on the same data and combine their final predictions to generate a better overall prediction accuracy. Different ML methods are more suitable to different types of data and so some outperforms others on certain tasks. Combining them in a weighted manner can therefore take advantage of different methods in different situations. This approach does not deduce or use the structural features of the data.

The critical difference of our approach is to extract an intrinsic property, the structural feature, of the system (based on data). This structural feature is then used to augment the downstream analysis, regardless of the algorithms used.

These two approaches are complementary. For instance, one can continue to use ensemble predictions, after imposing the structural constraint.

We have revised our discussion to be more precise and concrete.

9. On pages 24 and 25, I would suggest changing “transforms the data into a linearly separable space” in a way that reflects the fact that linear separability is a property of two sets of points not of a space.

We thank the reviewer for catching this important technical detail and we have changed accordingly in the Methods section.

10. On pages 25 and 26, the authors state “a soft constraint is applied to the additional feature from classification, f(p’), by letting the regression method learn an optimal weight w for it [...]”. As far as I understand the weight w, is a hyperparameter that is set using cross-validation. I advocate reserving the word learn for parameters that are set by the learning algorithm and not during hyperparameter tuning.

We thank the reviewer for catching this conceptual detail as well and we have replaced the word “learn” with “choose,” which is a more conventional term for hyperparameter tuning.

**Reviewer #2:**

The manuscript by Ha and colleagues describes a new machine learning approach, called structure-augmented regression (SAR), that captures phenotypic or fitness landscapes using less data than traditional regression approaches. In the paper they compare their approach to polynomial regression, support vector regression, random forest and k-nearest neighbors. They apply the approach to different data sets of increasing complexity.

Overall, I think that this is an interesting approach that could have wide applicability. I have some suggestions/comments for the improvement of the manuscript.

We thank the reviewer for the thorough review of our paper, for finding it interesting and recognizing its wide applicability. We appreciate all the constructive comments and suggestions, which we address thoroughly below. We have also revised the manuscript accordingly.

**Major comments:**

1. What is the relationship of this approach to traditional design of experiments (DOE) and Bayesian optimization. While I understand that the goal may be slightly different, they are both approaches used widely. Bayesian Optimization also estimates the fitness landscape, for example. Some text should be added in the introduction.

Our method is complementary to these two methods.

Indeed, Design of Experiments (DOE) is also a methodology that investigates the effect of combinations of relevant factors on the biological system of interest. Specifically, during DOE, for each factor, a specific set of values to be tested are first determined from some previous screenings or available literature data, then these values are combined in a (full or partial) factorial manner^9^. In contrast, in our methodology, we could randomly select the values for each factor and then randomly combine them instead of in a factorial manner. This is again due to fact that the key to our method is to estimate a structure of the underlying data first from classification. Therefore, as long as the randomly generated combination returns different classes, such structure can be estimated to various degrees. This is also how we tested our methods across different examples, i.e., we first randomly pick a subset from all data as the training set, then test the learned model on the rest of the data. Therefore, in comparison to DOE, our method is less stringent both in data generation and previous knowledge of the system.

Bayesian optimization, as its name suggested, has a specific objective function to optimize. During this process, indeed, Bayesian optimization learns an estimated landscape too. The key difference between Bayesian optimization and our method is that our method does not specifically aim to optimize any function, i.e., find the maximized location of the landscape. Instead, our method aims to estimate the whole landscape.

We have added this comparison in the paragraph where we discuss possible next steps for this method in the Discussion section.

2. I was confused about the use of direct regression and SVR. While I did eventually figure this out, you should make it clearer to reader as sometimes you use direct regression and sometimes SVR, meaning the same thing. It wasn’t clear what the distance means here. I think you mean distance from the decision boundary.

We regret the confusion.

We made the text clearer by stating that the direct regression method is SVR in page 7. Here we attach the exact phrase ““We find that our soft-constraint regression method is superior in the same prediction task, compared with direct regression (SVR alone).” We also stated that distance means the distance from the learned classification boundary explicitly on the same page.

3. Is there an issue with using the data set twice, once for classification and once for regression? I guess if you had enough data you could do these on two samples of the training data points. This could be discussed somewhere.

We agree with the reviewer that with enough data, one can do the two learnings on different samples of the training data points.

We also appreciate the reviewer’s astute comment on our method using the data twice. Given limited data, this practice is in fact critical to our approach. The two steps serve two different purposes. The classification step teases out the structural feature of the landscape. For a 3D landscape (two inputs, one output), the extracted boundary represents its 2D projection, upon specifying a threshold value in the output. The key to our regression step is by imposing this structural constraint, in guiding the subsequent regression. As a result, our regression makes an implicit assumption: equal distances from the boundary correspond to approximately the same values. The standard regression method does not apply this assumption.

We have revised our text to better clarify this point.

**Minor comments:**

1. Pg6. Figure 1C is referenced after D,E

We thank the reviewer for their thoroughness. We have changed the Figure 1 accordingly so that the panels in it are referenced in sequence.

2. Pg12 “in comparison to the counterpart”

We thank the reviewer for pointing this out. We have changed the text accordingly.

**Reviewer #3:**

This paper describes a machine learning methods for bioengineering purposes. The innovation is the utilization by the method of the underlying structure of the biological system under investigation. This algorithm is used to infer the phenotypic response of the system under perturbation. The authors claim that the algorithm can be used to augment a synthetic biology platform and allow for faster exploration of built biological structures and combinatorial experiments for drug design.

This is a very insightful paper, which postulates the existence of a learnable (usually lower dimensional) manifold for input-output relationships in biological systems. The manifold itself is built from data by assuming there is lower dimensional boundary differentiating distinct phenotypes, and then by letting the algorithm discover it.
I like this paper, but I think there are a few shortcomings which should be addressed during revision.

We thank the reviewer for the thorough review of our paper, for finding it insightful and recognizing the key conceptual innovation. We appreciate all the constructive comments and suggestions, which we address thoroughly below. We have also revised the manuscript accordingly.

**Major comments:**

1. Firstly, the definition of "structure" is vague. I interpret is as the existence of a complex, potentially nonlinear mapping between the inputs and outputs of a biological process. The authors do not give a formal definition. as far as I can tell, but rather leave to the readers the interpretation of this fundamental concept. I think the authors should explain more formally, concretely and above all explicitly what the structure of the biological system is, since it is so central to their methodology.

We thank reviewer for pointing this potential confusion out. Indeed, the definition of “structure” is at the core to our methodology.

By structure, we mean there is an overall regular trend of the biological response that exists on a (potentially) high-dimensional landscape that can be complex and nonlinear as the reviewer pointed out. While this mapping between the inputs (e.g. systematic disturbances like drug combination treatments) and outputs (e.g. system response like cell growth under these treatments) can be potentially complex and nonlinear, there is an overall regular trend of the response, i.e., the overall response is not random on the original input space. Moreover, in a lot of biological systems, this regular trend can be well-captured by two pieces of lower-dimensional information. The first piece of information is the lower-dimensional boundary in between distinct classes (e.g. phenotypes). The second piece of information is that the regularity in the response trend can be captured by assuming that same distance from the datapoints to the boundary implies approximately similar responses.

The uniqueness of our methodology is that while explicit modeling such response requires a priori knowledge of the model formulation, thus, the input-output relationship, our method does not assume any specific input-output formulation. It only takes advantage of the structural information, i.e., the low-dimensional boundary and the regularities in the response trend, thus is more generalizable and flexible, and as the multiple figures show, requires little data to predict with a high accuracy.

We have also expanded the Introduction section to explain this “structure” idea accordingly (the third and second to last paragraph in the introduction section).

2. The authors use support vector classification for their boundary discovery, but state that any regression-based classification method would be sufficient. Given the importance of this step, I would like to see more thorough discussion about it, with the limitations embedded in the various choices of algorithm. Right now, this is limited to the appendix, and I would prefer to see it in the main text.

We thank the reviewer for pointing this important concept out.

For the classification method to work in our pipeline, it needs to satisfy two criteria, namely, it needs to return a boundary as well as a function to calculate a value that’s positively correlated with the distance between a point and the boundary. For each point. SVC is a fitting classification method since it returns a vector-based function, with which one can calculate the distance between each point and the learned boundary.

Indeed, there are other regression-based classification that satisfies these two criteria, one of the most used ones is logistic regression classification. This classification method also returns a function (a logistic function) after learning to classify. We can therefore use this logistic function as a proxy for our distance function as well. We include the same analysis here and inserted this result as our Supplementary Figure 5. One can see that indeed, using the logistic regression classification in the first step of the pipeline, it works consistently across different examples as well.

As to the limitations embedded in the various choices of algorithm, we do not have an intuitive explanation on the observed difference based on the inner workings of different algorithms. However, it appears that the difference might result from the baseline performance of an algorithm on limited data. If the regression by a particular algorithm performs poorly, there is more room for further improvement, either by generating more data or by using structure-augmented regression. However, if an algorithm is already performing well, incorporation of the structural information may not help as much.

For example, in the third example (third row), random forest regression alone performs the worst across all four regression methods, and structure-augmented random forest regression shows the most improvement. However, structure-augmented kNN and structure-augmented polynomial regression are on par with the structure-augmented random forest regression, yet the improvement is not as significant since there isn’t much to improve for these two methods. On the other hand, in the fourth example (last row), random forest regression alone performs very well already, so while structure-augmented random forest regression still improves the performance, there is not much room to improve. Meanwhile, all other three regression methods alone seem to perform worse, so structure-augmented SVR, structure-augmented kNN and structure-augmented polynomial regression all show much more significant improvement.

Likewise, similar trends exist when using SVC as the classification method (Supplementary Figure 4).

Specifically, in the third example (third row), random forest regression alone performs the worst across all four regression methods, and structure-augmented random forest regression shows the most improvement. However, structure-augmented kNN and structure-augmented polynomial regression are on par with the structure-augmented random forest regression, yet the improvement is not as significant since there is not as much to improve for these two methods. On the other hand, in the fourth example (last row), random forest regression alone performs very well already, so while structure-augmented random forest regression still improves the performance, there is not much room to improve. Meanwhile, all other three regression methods alone seem to perform worse, so structure-augmented SVR, kNN, and polynomial regression all showed more significant improvement.

**Minor changes:**

1. The abstract contains a reference to SVR, which has not been introduced. Is that a typo and the authors mean SAR instead?

We thank the reviewer for identifying this significant typo and we fixed it.

2. Going into the appendix, one is left with more questions than answers by reading the Structure-Assisted Regression section, which is not written having a general application in mind, but rather for an example of cell survival for a drug combination experiment. Is it possible to apply this method in general? What would be the function that someone would need to use to do that?

We thank the reviewer for pointing out the confusing parts in the Structure-Assisted Regression section.

It is a method that’s designed to be applied in general. We wrote with a concrete example in mind to be less abstract and with less emphasis on the math and more emphasis on the flow of the method.

We acknowledge that this writing could create the impression that this method is not generally applicable.

We have added in sections to explain how it can be applied to other examples in general.

3. In general, the use of the various acronyms (SVC, SAR, SVR) is very confusing. Can anything be done about that?

We agree that using three terms together can be confusing. We have put the full name of these 3 acronyms in figures where they appear together or in pair to make them less confusing.

**Reviewer #4:**

In the manuscript titled “Data-driven learning of structure augments quantitative prediction of biological responses”, Ha et al. proposed a new method of incorporating structural information into regression to predict outcome multi factor induction. The critical contribution is this method would greatly reduce the amount of data needed. I found the results quite interesting and presentation thorough, with some clarification questions:

1. As I am trying to understand the method, I found Fig. 1C is a bit confusing (the caption lacks some key details). First, the solid black line is really hard to identify. It took me a while to see, please make that line easier to locate.

We regret the confusion. We increased the width of the solid black line and added in some additional details to the figure caption and the main text (page 7).

2. Second, in Fig. 1C right panel, how the “calculated distance” is calculated?

The calculated distance is calculated from the equation returned by the first classification step of the pipeline.

Specifically, this equation returns approximately same value for each point that is approximately the same distance from the boundary.

We explain this equation and the calculation in detail in the Methods section. We added a note to point readers to the Methods section to these details as well.

Please see attached below:

“

The outputs of SVC include both the predicted labels and a distance function:

Predicted label of cell survival versus death: Ypred = [y_pred1_,…,y_predi_,…,y_predn_]. (9)

The distance function: $f\left( \boldsymbol{p} \right)= \sum_{i} \alpha_{i}y_{i}K<p_{i},p>+ \lambda_{0}$,	(10)

yi and pi are input values for observation i. αi is the weight of observation i, and λ0 is the bias term. K here represents a kernel function that transforms the input data into a new space where the transformed data is now linearly separable by SVC in that space. Both the parameters αi and λ0, and the kernel, as well as the kernel hyperparameters are optimized by the SVC algorithm. Specifically, we let SVC choose the best kernel from linear, polynomial and RBF kernels. Likewise, the kernel hyperparameters, i.e., the degree and the coefficient term for the polynomial kernel, and the gamma term for both polynomial and RBF kernels are all optimized with cross-validation. We used python’s scikit packages for all training and optimization.

”

3. What does “ground truth” mean?

When training and testing our machine learning models, ground truth means the experimentally measured final density of S1 under different environmental conditions/drug combinations. They are the gold standards used to evaluate and compare the models.

During training, ground truth is given to the machine learning algorithm to learn a model; during testing, ground truth is used to evaluate the model performance when predicting on unseen data.

4. The last paragraph on page 6 needs some clarification. All the later figures use prediction and ground truth as axis labels with both scale 0 to 1. How come Fig 1C right panel x-axis range -200 to ~100, with a name calculated distance? Maybe I am missing something here. I would expect a scatter plot of predicted value VS simulated value (ground truth), and compare it to a straight line, thus get a R2 value.

We regret the confusion.

Indeed, a wide range from -200 to ~100 can be confusing as the first example to illustrate the “calculated distance” concept. We have chosen another example that has a smaller range, which hopefully serves as a more intuitive example to explain this concept.

Here we explain what the calculated distance means.

The calculated distances in Fig 1C are not the final prediction that correspond to the ground truth, which fall into the range of 0 to 1.

As the first paragraph on page 7 explains and the Method section expands on, the first step of SAR is a classification task, which returns a boundary between classes and a function that we use to calculate the “distance” between each datapoint to the classification boundary. Therefore, this intermediate panel has the name “calculated distance.” This calculated distance can take on both positive and negative values. It is negative on one side of the boundary and will be positive on the other side. More specifically, the further the point is from the boundary, the larger the absolute magnitude this value is.

Therefore, this distance provides useful information for the regression, as it captures the overall landscape trend.

This “calculated distance” then serves as an extra feature for the regression step.

The top row of Fig 1D, Fig 1E and F illustrates the whole flow.

5. Fig 2 C and F bottom right panel show some results as expected. But then in Fig3 the results do not look so good. How come Fig 1G right bottom panel results look like a sigmoidal curve?

The purpose of this method is to increase the prediction accuracy given limited datapoints to learn from. To demonstrate this point, we compare its performance to those of the traditional ML regression methods, on both simulation results (Fig 2) and experimental results (Fig 3).

Fig 2 are simulation results. Fig 3 are experimental results, which have landscapes that are noisier, so the overall prediction accuracy using either pipeline (SAR or SVR) decreases when applying on Fig3. However, it’s still the case the SAR performs significantly better.

Fig 1 is to demonstrate the SAR’s advantage of having a good prediction accuracy even when the training data is sparse. Therefore, we only used 10 datapoints as the training set, to make the point. Given only 10 datapoints, we do not expect a perfect prediction, i.e. a linear correlation on scatter plot. However, SAR does show significant improvement over SVR, which is clear from the 0.96 R^2^ value of SAR and the 0.81 R^2^ value of SVR.

**References:**

1. Chih-Wei Hsu, & Chih-Jen Lin. A comparison of methods for multiclass support vector machines. *IEEE Transactions on Neural Networks, 13(2)* (2002).
2. Duan, K.-B., & Keerthi, S. S. Which is the best multiclass SVM method? an empirical study. *Multiple Classifier Systems, 278-285* (2005).
3. Lopatkin, A. J., Huang, S., Smith, R. P., Srimani, J. K., Sysoeva, T. A., Bewick, S., Karig, D. K., & You, L. Antibiotics as a selective driver for conjugation dynamics. *Nature Microbiology*, *1*(6) (2016).
4. Lopatkin, A. J., Meredith, H. R., Srimani, J. K., Pfeiffer, C., Durrett, R., & You, L. Persistence and reversal of plasmid-mediated antibiotic resistance. *Nature Communications*, 8(1) (2017).
5. Wang, T., & You, L. The persistence potential of transferable plasmids. *Nature Communications*, 11(1) (2020).
6. Burr Settles. Active Learning Literature Survey. *Computer Sciences Technical Report 1648, University of Wisconsin–Madison*. 2009.
7. Osugi, T., Deng Kun, & Scott, S. Balancing exploration and exploitation: A new algorithm for active machine learning. *Fifth IEEE International Conference on Data Mining* (ICDM’05).
8. Cohn, D., Atlas, L., & Ladner, R. Improving generalization with active learning. *Machine Learning*, 15(2), 201–221 (1994).
9. Durakovic, B. Design of experiments application, concepts, examples: State of the art. *Periodicals of Engineering and Natural Sciences (PEN)*, 5(3) (2017).
